# A mechanistic evolutionary model explains the time-dependent pattern of substitution rates in viruses

**DOI:** 10.1016/j.cub.2021.08.020

**Published:** 2021-11-08

**Authors:** Mahan Ghafari, Peter Simmonds, Oliver G. Pybus, Aris Katzourakis

**Affiliations:** 1 Department of Zoology, University of Oxford, Oxford, UK; 2 Nuffield Department of Medicine, University of Oxford, Oxford, UK

**Keywords:** paleovirology, molecular clock, substitution rate, mutation rate, substitution saturation, site saturation, time-dependent rate phenomenon, coronavirus, hepatitis C virus, foamy virus

## Abstract

Estimating viral timescales is fundamental in understanding the evolutionary biology of viruses. Molecular clocks are widely used to reveal the recent evolutionary histories of viruses but may severely underestimate their longer-term origins because of the inverse correlation between inferred rates of evolution and the timescale of their measurement. Here, we provide a predictive mechanistic model that readily explains the rate decay phenomenon over a wide range of timescales and recapitulates the ubiquitous power-law rate decay with a slope of −0.65. We show that standard substitution models fail to correctly estimate divergence times once the most rapidly evolving sites saturate, typically after hundreds of years in RNA viruses and thousands of years in DNA viruses. Our model successfully recreates the observed pattern of decay and explains the evolutionary processes behind the time-dependent rate phenomenon. We then apply our model to re-estimate the date of diversification of genotypes of hepatitis C virus to 423,000 (95% highest posterior density [HPD]: 394,000–454,000) years before present, a time preceding the dispersal of modern humans out of Africa, and show that the most recent common ancestor of sarbecoviruses dates back to 21,000 (95% HPD: 19,000–22,000) years ago, nearly thirty times older than previous estimates. This creates a new perspective for our understanding of the origins of these viruses and also suggests that a substantial revision of evolutionary timescales of other viruses can be similarly achieved.

## Highlights

• Chronological saturation of sites creates a power-law rate decay with slope −0.65
• Our model can be used to correctly estimate the rate decay and viral ages
• Humanity may have been exposed to sarbecoviruses since the Paleolithic period
• The origins of HCV date back before human migration out of Africa

## Teaser

Ghafari et al. develop a predictive mechanistic model that explains the evolutionary process behind the time-dependent rate phenomenon across timescales in viruses. The diversification of hepatitis C virus genotypes dates back to ∼423,000 years before present, and the most recent common ancestor of sarbecoviruses dates back to ∼21,000 years ago.

## Introduction

The timescale over which viruses evolve and how this process is connected to host adaptation has been an area of considerable research and methodological progress in recent decades. Mammalian RNA viruses, in particular, exhibit extraordinarily rapid genomic change,^1^, ^2^, ^3^ and analyses of their genetic variation have enabled detailed reconstruction of the emergence of viruses such as HIV-1,^4^ hepatitis C virus,^5^ and influenza A virus.^6^ RNA viruses display evolutionary change over short timescales (weeks to months) and can alter a substantial part of their genomes following a host switch.^7^, ^8^, ^9^, ^10^ Well-characterized examples for both RNA and DNA viruses include the emergence of HIV-1 in humans from a chimpanzee reservoir^4^^,^^11^ and the adaptation of myxomatosis in rabbits.^12^

These rapid rates of virus sequence change stand in striking contrast with evidence for extreme conservation of virus genome sequences over long periods of evolution and at higher taxonomic levels. Inferred short-term rates of virus sequence change should create completely unrecognizable genome sequences if they were naively extrapolated over thousands, or even hundreds, of years, yet endogenous viral elements (EVEs) that integrated into host genomes throughout mammalian evolution are recognizably similar to contemporary genera and families of *Bornaviridae*, *Parvoviridae*, and *Circoviridae*, among many other examples.^13^, ^14^, ^15^ This observation is complemented by evidence from studies of virus/host co-evolution^16^, ^17^, ^18^ and, more recently, from analyses of viruses recovered from ancient DNA and RNA in archaeological remains,^19^, ^20^, ^21^, ^22^ which indicate a remarkable degree of conservation in viral genome sequences and their inter-relationships at genus and family levels. This dichotomy has been attributed to the time-dependent rate phenomenon (TDRP), which is the observation that apparent rates of evolution are dependent on timescales of measurement.^23^^,^^24^

The TDRP has been explained by processes such as sequence site saturation, purifying selection, short-term changes in selection pressure, and potential errors in the estimation of short-term substitution rates.^23^, ^24^, ^25^, ^26^ Empirically, substitution rates across RNA and DNA viruses show a striking linear relationship between log-transformed rates and timescales of measurement, despite the large variation among viruses in their initial short-term substitution rates.^25^ The regression gradients of observation time against estimated evolutionary rates are consistently around −0.65 for all virus groups in which long-term substitution rates can be calculated or inferred. Crucially, the observation of a universal power-law rate decay in nearly all viruses suggests that there is a common underlying evolutionary process. However, we do not have a systematic biological explanation for this observation. Various factors have been invoked to account for these patterns, including purifying selection, site saturation, and sequencing errors, though none of these alone have been shown to generate a power-law rate decay.^24^^,^^26^

One proposal is that the primary driver of virus evolution over long evolutionary timescales is host adaptation, in which virus sequence change is severely curtailed by stringent fitness constraints.^27^ Viruses exist within a tightly constraining host niche to which they rapidly adapt; paradoxically, their high mutation rates, large population sizes, and consequent ability to adapt rapidly serve to restrict their long-term diversification and sustained sequence change, rendering them evolutionary “prisoners of war” (PoWs). This idea posits that, over longer timescales, rates of viral evolution will be bound by the rate of evolution of their hosts.^27^ However, the exact timescales over which these various evolutionary events occur, as well as the extent to which they contribute to changing virus sequences over time, is still largely unknown.

Here, we develop a new model of the longer-term evolutionary rate dynamics of viruses that explains the empirical observation of a universal power-law rate decay across different virus groups. Our model is biologically motivated and based on a minimal number of assumptions. We show how the rapid genetic saturation of some sites, together with the host constraint on other sites, can create a time-dependent rate dynamic whereby sites can partially or fully saturate according to how fast they accumulate substitutions over time. This process occurs chronologically from sites evolving the fastest to those that evolve epistatically, to those that evolve at the hosts’ substitution rate. This model reproduces empirically observed TDRP patterns, and the inflection points where time-dependent rate changes become manifest due to site saturation. We demonstrate that the model predictions are robust to intrinsic and marked differences in substitution rates among different virus groups, and to assumptions about the relative proportion of sites evolving at different rates.

## Results

### Power-law rate decay can emerge due to site saturation

First, we show how a time-dependent rate effect emerges when estimating the rate of sequence divergence using a standard evolutionary model. Suppose that a sequence has diverged from its ancestor for t years under a constant and uniform substitution rate per site per year (SSY), μ. The proportion of pairwise differences between the derived sequence and its ancestor, p(t), initially accumulates linearly (i.e., p(t)≈μt) until it reaches a point where every new substitution occurs in the background of a site that has already changed at least once; this limit, hereafter called the saturation frequency, α, occurs at time $t^{*}\approx \alpha /\mu$ (Equation 1). As the derived sequence continues to diverge beyond the saturation point, $t^{*}$, the observed proportion of pairwise differences, $\hat{p}$, remains effectively unchanged. If there are no intermediate samples from the derived sequence before it reaches the saturation point, any conventional substitution model is not able to correctly count the number substitutions and, therefore, underestimates the true rate. Thus, without enough temporal information from the derived sequence before reaching the saturation point, using any conventional substitution model, the inferred genetic distance (i.e., the expected number of substitutions per site), $\hat{d}$, remains constant and the estimated substitution rate, $\hat{\mu }$, declines as the divergence time increases, $\hat{\mu }\propto 1/t$, which manifests itself with a power-law rate decay with slope −1 on a log-transformed plot.

### Conundrum of rate calibrations

As an illustrative example, we consider how time-dependent rate effects pose a challenge to the estimation of the substitution rate of foamy viruses (FVs) over time. FVs are a group of retroviruses in the subfamily of *Spumaretrovirinae* that have been isolated from a broad range of mammals.^28^ By tracking the evolution of FVs in a population of African green monkeys over short timescales (i.e., less than a decade), it was estimated that their evolutionary rate is approximately $3.8\times 10^{-4}$ SSY.^29^ By contrast, using the very long and stable cospeciation history of FVs with their hosts, which goes back more than a hundred million years,^17^ their long-term rate of evolution has been estimated to be nearly $1.7\times 10^{-8}$ SSY,^30^ almost four orders of magnitude slower than their short-term rates.

**Figure 1:**
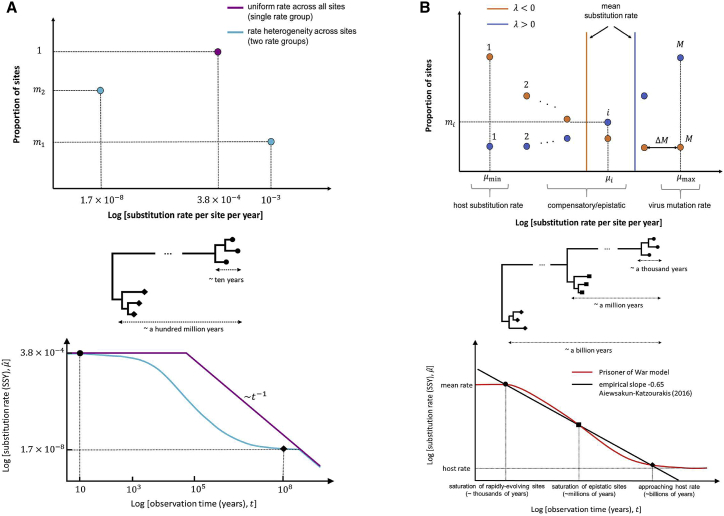
Time-dependent rate phenomenon in foamy viruses (FVs) under different evolutionary models (A and B) How time-dependent rate phenomenon emerge (A) using a standard evolutionary model with a single or double rate group and (B) using the PoW model with several rate groups. (A) Distribution of the fraction of sites per rate group when (top) there is a single rate group (purple), i.e., all sites evolve at the same substitution rate, or there are two rate groups (blue) such that a fraction, $m_{1}=0.38$, are evolving at a faster rate ($10^{-3}$SSY) compared to the remaining sites, $m_{2}=1-m_{1}$, which are evolving more slowly ($10^{-6}$SSY). (B) Top: there are M rate groups that are equally spaced on a log-scale, with a common ratio, ΔM, from the slowest rate (group 1), $\mu_{\min}=10^{-9}$SSY, to the fastest (group M), $\mu_{\max}$. According to the PoW model, a fraction of sites, $m_{i}$, belonging to rate group i∈{1,2,…,M}, evolving at rate $\mu_{i}$, is an exponentially distributed number with exponent parameter λ such that λ is less (greater) than zero when the majority (minority) of sites evolve at the host substitution rate. (A, bottom) Schematic plot of the evolutionary rate trajectory of FVs over time, assuming that their rate is inferred using a standard substitution model with a single rate group that is calibrated based on their short-term substitution rates (circular nodes on the tree) and two rate groups based on their mean and long-term substitution rates (diamond-shaped nodes on the tree). (B, bottom) Evolutionary rate trajectory of viruses over time under the PoW model, whereby the short-term rates can be inferred until when the fraction of sites belonging to the fastest rate group reaches saturation (the inflection point in the curve), beyond which point a time-dependent rate decay emerges. The virus substitution rates can be reliably inferred across all timescales without a need for any rate calibrations and the pattern of rate decay is aligned with the empirical slope of −0.65.^25^ Related to STAR Methods. See also Figure S1.

If we calibrate the amount of divergence (i.e., the expected number of substitutions per site) between FV sequences over time using their short-term evolutionary rate estimates, then nearly all sites in the virus genome should have acquired a substitution (i.e., saturation point) after ∼2,500 years. Beyond this time, any standard model of sequence evolution (substitution model) underestimates the amount of sequence divergence and, thus, the inferred substitution rates, $\hat{\mu }$, drop sharply as the time span of observation, t, increases such that the slope of the rate decay on a log-transformed plot is −1 (Figure 1A).

Even though there is a sharp decline in the inferred substitution rate of FVs over time due to site saturation, it still overestimates their true long-term evolutionary rate based on cospeciation history after a hundred million years (Figure 1A, purple line). One way to resolve this is by dividing the sites in the FV genome into two “rate groups,” such that a fraction of sites, $m_{1}$, evolve at a fast rate (say, $\mu_{1}=1\times 10^{-3}$ SSY), and the remaining sites, $m_{2}=1-m_{1}$, evolve at a slower rate (say, $\mu_{2}=1.7\times 10^{-8}$ SSY). The allocated fraction, $m_{1}$, can be specified such that the mean substitution rate, $\left\langle \mu \right\rangle =m_{1}\mu_{1}+m_{2}\mu_{2}$, is equal to the observed short-term evolutionary rate of FV, $\overset{⌢}{\mu }=3.8\times 10^{-4}$ SSY. Therefore, a standard substitution model can reliably estimate short-term rates up to and before the saturation of rapidly evolving sites, $m_{1}$, beyond which time the number of substitutions at those sites is underestimated and a time-dependent rate decay emerges. However, over longer timescales, new substitutions at the slowly evolving sites, $m_{2}$, accumulate such that the inferred rate, $\hat{\mu }$, gradually plateaus at a rate which corresponds to the long-term substitution rate of FV, $\mu_{2}$. This new plateau will only last until the proportion of slow-evolving sites also saturate, which takes more than 100 million years to occur, beyond which time the entire genome is saturated and a power-law rate decay with slope −1 emerges (Figure 1A, cyan line).

### The PoW model of virus evolution

Even though an evolutionary model for the FV substitution rate based on two rate groups can recover its inferred short-term and long-term rates, it still fails to accurately predict the observed substitution rates over intermediate timescales. Aiewsakun and Katzourakis^31^ made the empirical observation that the time-dependent rate of FVs follows a power-law decay with a slope of −0.65. More importantly, they showed that not only FVs but all other virus groups for which a correlation could be performed follow a similar universal power-law rate decay with the same slope,^25^ suggesting a common underlying process involved in rate decay.

The PoW model of evolution is based on the principle that a virus sequence is divided into M substitution rate groups, ranging from sites evolving very rapidly at rate $\mu_{\max}$, similar to virus mutation rates, to sites evolving more slowly due to epistatic and compensatory substitutions, all the way to sites evolving at the host substitution rate, $\mu_{\min}$. The fraction of sites, $m_{i}$, allocated to each rate group, i, is an exponentially distributed number, $m_{i}=Ce^{\lambda i}$, where C is the normalization factor and λ is the exponent coefficient that determines whether the majority of sites are slowly (λ<0) or rapidly (λ>0) evolving. As the virus sequence evolves, sites belonging to the fastest rate group saturate first, typically after $t∼1/\mu_{\max}$ years, followed by the saturation of sites in the next fastest rate group, which takes longer to occur, and so on. Depending on how divergent a virus sequence is with respect to its ancestor, sites that belong to more slowly evolving rate groups may also reach partial or complete saturation (Equation 4). Thus, at any given time span, the virus explores only a subset of its sites (i.e., is trapped inside a “prison cell” in sequence space) and does not have access to explore substitutions at every position in its genome. This chronological saturation of sites gives rise to a power-law decay in the inferred substitution rate over time with a slope of −0.65 on a log-transformed graph, supporting the empirical observation by Aiewsakun and Katzourakis (Figure 1B).^25^

Building upon a collection of virus evolutionary rate estimates from more than 130 publications,^25^ we use 396 nucleotide substitution rate estimates across six major viral groups to find the line of best fit between the PoW model of time-dependent substitution rates and the evolutionary rate estimates for each viral group (i.e., the data that are collected from the literature) using the geometric least-squares method.^32^ This enables us to estimate a mean and maximum substitution rate (i.e., ⟨μ⟩ and$\mu_{\max}$) for each virus group.

**Figure 2:**
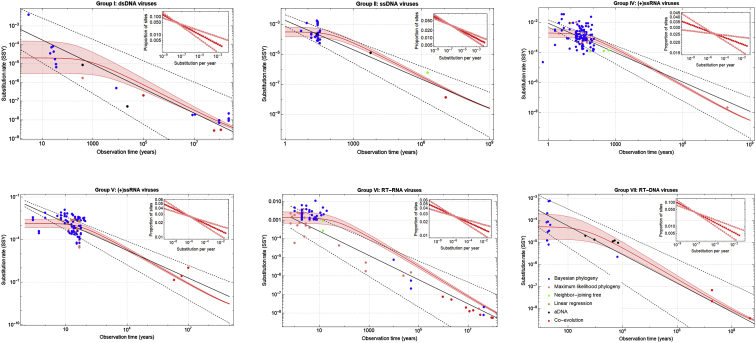
Estimated time-dependent rate curves for each virus group according to the PoW model A total of 389 viral rate estimates (colored circles representing various phylogenetic methods used for rate estimation) was collected from more than 130 publications: 23 estimates for Baltimore group I, 32 for group II, 123 for group IV, 106 for group V, 85 for group VI, and 20 for group VII. The insets show the proportion of sites in each rate group. Every rate group, including the ones with the smallest proportion, are well-represented in the genome, i.e., $m_{\text{minimum}}\gg 10^{-4}>1/L$, where L is the typical genome size of an RNA virus. The red lines show the best fit and shaded area the 95% confidence intervals for each virus group (ΔM=1.58and $\alpha_{M}=\alpha =3/4$). See also Figure S2.

Our results show that upon the saturation of the fastest-evolving sites a power-law rate decay emerges that is in agreement with the empirical slope −0.65 (95% HPD: −0.72, −0.52) across all viral groups (Figure 2). The inflection point in the rate curve, which signals the saturation of rapidly evolving sites, occurs typically after 100 to 1,000 years in most RNA and DNA viruses. We further find that in double-stranded DNA (dsDNA) viruses, the short-term substitution rate (i.e., the flat part of the time-dependent rate curve, $\left\langle \mu \right\rangle =\sum_{i=1}^{M}m_{i}\mu_{i}$) and the fastest-evolving rate group, $\mu_{\max}$, have the lowest rates compared to all other virus groups. Together with reverse-transcribing DNA (RT-DNA) and single-stranded DNA (ssDNA) viruses, dsDNA viruses typically have 1 to 2 orders of magnitude slower short-term substitution rates and fastest-evolving rate groups compared to RNA viruses (Table 1). Conversely, these rates are very similar among the positive-strand RNA (+ssRNA) viruses, negative-strand RNA (–ssRNA) viruses, and reverse-transcribing RNA (RT-RNA) viruses. We find that the estimated rate at the fastest-evolving sites in RNA viruses is $\mu_{\max}\approx 4\times 10^{-2}$SSY, which is very close to their estimated mutation rates. Therefore, these sites can begin to saturate after only a few decades or hundreds of years. On the other hand, we find that a large proportion of slow-evolving sites are not saturated over the span of more than 1 billion years and that the rate curve in none of the virus groups has completely plateaued at the host substitution rate (Figure 2).

**Table 1:** Estimated short-term and maximum substitution rate SSY according to the PoW model

Viral group	Type of virus	Short-term substitution rate, ⟨μ⟩	Fastest rate group, $\mu_{\max}$
I	dsDNA virus	$2\left(0.3-16\right)\times 10^{-5}$	$3\left(0.6-10\right)\times 10^{-3}$
II	ssDNA virus	$3\left(1-6\right)\times 10^{-4}$	$2\left(1-3\right)\times 10^{-2}$
IV	(+)ssRNA virus	$2\left(1-4\right)\times 10^{-3}$	$4\left(3-6\right)\times 10^{-2}$
V	(−)ssRNA virus	$1\left(0.7-3\right)\times 10^{-3}$	$4\left(3-6\right)\times 10^{-2}$
VI	RT-RNA virus	$1\left(0.7-3\right)\times 10^{-3}$	$4\left(3-6\right)\times 10^{-2}$
VII	RT-DNA virus	$5\left(1-20\right)\times 10^{-5}$	$4\left(2-10\right)\times 10^{-3}$

The inferred short-term substitution rate and the rate of substitution at the fastest-evolving rate group across six virus groups. Numbers in parentheses show the 95% confidence interval for an estimated paramater. See also Table S1.

To ensure that our model predictions are not biased toward a particular virus family with more evolutionary rate estimates (i.e., more data points to fit to the PoW model in Equation 4), we remove all the short-term rate estimates (i.e., any rate that is measured over a time span of less than 100 years) within each viral group except for one virus family or genus to recalibrate the mean substitution rates (Table S1). We find that, despite the broad variation in short-term rates across all viral groups, the shape of the sigmoid curve, exponent coefficient λ, and $\mu_{\max}$is robust to such changes and is not an artifact of systematic biases in selecting rate estimates from a particular virus family. We note that, in group VI, the large difference in evolutionary rates between *Lentivirus* and *Deltaretrovirus* families results in a noticeably different pattern of rate decay over short timescales (Figure S2) and the long-term rates are more aligned with the predictions based on the *Deltaretrovirus* recalibration. The short-term substitution rates of *Lentivirus* families are 1 to 2 orders of magnitude higher than the *Deltaretrovirus* family. The latter evolves at rates similar to RT-DNA viruses. We also see that a larger fraction of sites in DNA viruses tend to evolve at rates closer to the host substitution rates (i.e., the majority of sites in the virus sequence are slow evolving) compared to RNA viruses, which largely have an equal proportion of sites in every rate group (i.e., the exponent coefficient λ is close to zero). We also carried out a similar sensitivity analysis at the level of virus genera, which further confirms that the rate curves predicted by the PoW model remain accurate at this level and are not artifacts of measured rates at the level of Baltimore groups (Figure S2).

**Figure 3:**
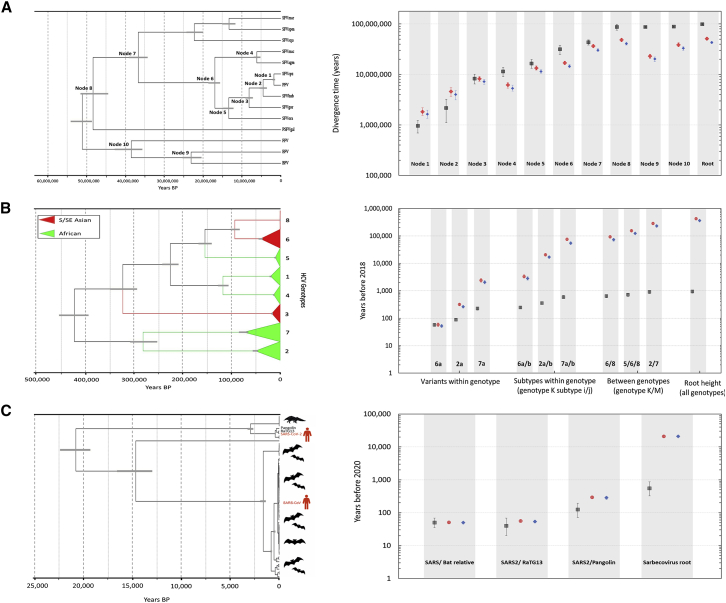
The PoW-transformed time-calibrated phylogenies and estimated divergence times for FV, HCV, and sarbecovirus datasets (A) FV phylogeny and estimated divergence times for labeled internal nodes using the PoW model with HKY (red) and JC69 (blue) substitution models and host calibrations (black).^31^ (B) HCV (including all 8 genotypes) phylogeny and estimated divergence times for variants within genotypes, subtypes, and between genotypes using the PoW model and a strict clock with HKY+G substitution model (black). (C) SARS-CoV-2 sarbecovirus phylogeny based on the non-recombinant alignment 3 (NRA3)^34^ constructed using the PoW model and a standard HKY+G substitution model with uncorrelated relaxed clock (black). Gray horizontal lines on the phylogeny and vertical lines on the graphs represent the 95% HPD. Related to STAR Methods and Figure S3.

The formulation of the PoW model allows for a one-to-one map between the relative genetic distance of any given pair of sequences and divergence time. Therefore, by estimating the genetic distance between sequences using some distance metric, we can convert them into divergence time (Equations 5 and 6). Given that the shape of the rate curve is robust to changes in the short-term substitution rates, ⟨μ⟩, across virus families and genera, the transformation from relative genetic distance to time can be applied to estimate the divergence times among any given virus sequences. To test the validity of this approach in recovering known divergence times, we re-estimate the time to the most recent common ancestor of various species of FVs and compare the results with estimates based on host calibrations.^31^ Because of the long history of cospeciation, the virus phylogenies have the same topology as the host. Therefore, there can be a one-to-one comparison between estimated divergence times based on their phylogenies. First, by fixing the rate of substitution at the fastest-evolving sites to those inferred for RNA viruses (Table 1) and using known long-term substitution rate estimates of FVs from the literature to find the line of best fit for the PoW rate curve, we estimate the FV short-term substitution rate to be 6.1 × 10^−6^ (95% HPD: 5.4 × 10^−6^ – 7.0 × 10^−6^), which is in agreement with previous estimates.^33^ We then construct a distance tree using the Jukes-Cantor (JC69) or Hasegawa, Kishino, and Yano (HKY85) model and convert branch lengths into divergence time using the PoW transformation (STAR Methods). The result confirms that we can reliably recover true divergence times between most samples (some are different by up to a factor of 2) without calibrating the dates of any nodes on the tree (Figures 3A and S3A).

To further illustrate the radical effect of applying the PoW model to virus evolutionary timescales, we analyze a heterochronous dataset of complete hepatitis C virus (HCV) genome sequences that represent its component genotypes and subtypes (Figure 3B). First, using a standard HKY+G substitution model, we find that the mean substitution rate of HCV is 8.3 × 10^−4^ (95% HPD: 7.3 × 10^−4^ – 9.5 × 10^−4^) SSY. Then, by using the predicted value of $\mu_{\max}$ for viruses that belong to group IV, $\mu_{\max}=3.65\times 10^{-2}$ (Table 1), and the inferred median short-term substitution rate $\left\langle \mu \right\rangle =8.3\times 10^{-4}$, we can construct a PoW-transformed time tree for HCV (STAR Methods). We find that there is a clear separation of timescales for the diversification of HCV variants within genotypes (∼50–2,000 years), among subtypes (∼1,000–80,000 years), and among genotypes (∼80,000–200,000 years) with an estimated time to the most recent common ancestor (TMRCA) of 423,000 (95% HPD: 394,000–454,000) years before present (BP) for HCV (Figure 3B). While the predicted divergence times for some of the within-genotype variants using the PoW model are similar to those obtained using a standard HKY+G substitution model, the latter estimates the TMRCA of HCV to be only 940 (95% HPD: 820–1,100) years BP with no clear separation of timescales for among-genotype diversifications. These results contrast with estimates of 500 to 2,000 years of genotype diversification by simple extrapolation from short term rates, while among-subtype divergence times of 1,000 to 80,000 years are up to 50 times higher than the 300 to 500 years estimated in previous molecular epidemiological analysis.^35^, ^36^, ^37^ The revised, very early evolutionary origin of HCV genotypes (394,000 years, 454,000 years 95% HPD) predicted by our model is striking. While these early dates still fit with proposed hypotheses for multiple and potentially relatively recent zoonotic sources of HCV in humans, associated with different genotypes,^38^^,^^39^ the existence of a common ancestor of HCV before human migration of Africa (150,000 BP) support an alternative scenario where HCV diversified within anatomically modern humans. HCV genotypes may have arisen from geographical separation in Africa (genotypes 1, 2, 4, 5, and 7) and migrational separation of human populations migrating out of Africa into Asia (genotypes 3, 6, and 8).

We also carried out a similar analysis to investigate the origins of the SARS-CoV-2 sarbecovirus lineage (Figure 3C). By finding the mean substitution rate of the sarbecovirus lineage to be 5.6 × 10^−4^ (95% HPD: 3.5 × 10^−4^ – 7.6 × 10^−4^) using a standard HKY+G substitution model (STAR Methods), we find that while the PoW-transformed phylogeny recovers the previous estimates for SARS-CoV and SARS-CoV-2 diversification from their most closely related bat virus over short timescales (i.e., less than hundreds of years BP), it extends the TMRCA back to 21,000 (95% HPD: 19,000–22,000) years BP, nearly 30 times older than previous estimates.^34^ The 95% HPD represents the uncertainty that may arise from the choice of substitution model, inferred genetic distance between each pair of sequences, and the inferred tree topology. Our results indicate that humanity may have been exposed to these viruses since the Paleolithic period if they had come into contact with their natural hosts. Also, our date estimates of the origin of the sarbecovirus lineage are in remarkable concordance with signatures of a selection of human genomic datasets that indicate an arms race with corona-like viruses dating back to 25,000 years BP,^40^ providing an external comparator for our methodology. We also note that, even if the sarbecovirus origins were estimated to be more recent, the pattern of selection could still be attributed to a deeper coronavirus ancestry.

To test the impact of changing the substitution model on the estimated divergence times, we compared our analyses on FV, HCV, and sarbecovirus datasets using both the JC69 and HKY substitution models and find minimal differences between the estimates (Figures 3 and S3). To further assess the impact of uncertainty in the clock model on estimated divergence times, we allow the short-term rates and $\mu_{\max}$ for each virus dataset to vary in accordance with its inferred posterior mean rate distribution and the geometric least-square cost function, respectively (STAR Methods). Our results showed that, while the median TMRCA estimates for the three datasets are very similar to results in Figure 3, the confidence intervals are much wider. We find that the TMRCA for the HCV dataset is 427,000 (95% HPD: 153,000–826,000) years BP and for the sarbecovirus dataset is 25,000 (95% HPD: 5,000–73,000) years BP (Figure S3). We note that the higher level of uncertainty in the estimated divergence times due to the clock model is somewhat artificial and can vary widely depending on the choice of the clock model (e.g., strict/relaxed clock) and rates prior. For instance, in the sarbecovirus dataset, because the alignments are from diverse viral populations with deep evolutionary histories, time-dependent rate effects become manifest over the span of 10 to 50 years of rate measurement. As a result, using an uncorrelated relaxed clock that allows each branch of the phylogeny to have its own evolutionary rate creates a very wide variation in the inferred mean substitution rate while the sigmoid shape of the time-dependent rate decay in the PoW model makes a very specific assumption about how the substitution rate varies as the timespan of rate measurement increases. Therefore, by allowing the short-term rate to vary in accordance with the posterior rate distribution, we can generate unwanted uncertainty in the PoW-transformed estimated divergence times.

## Discussion

The PoW model creates an over-arching evolutionary framework that can reconcile and incorporate timescales derived from co-evolutionary and ancient DNA studies. Further substantive re-evaluations of timescales of other RNA and DNA viruses using this approach may provide new insights into their origins and evolutionary dynamics. We show how these can alter paradigms about how we think that certain viruses evolved. The application of the PoW model will place ancestors of divergent virus sequences much further back into the past than conventional reconstructions. We obtained a good fit between the pattern of modeled and observed substitution rate decay gradients over time using only a minimal number of assumptions about mutational fitness effects and proportion of sites evolving at a particular rate. We showed that our method is robust to substantial differences in substitution rates among viral groups. By finding the short-term substitution rate (the flat part of the modeled rate decay) and the value of the fastest-evolving rate group (which sets the inflection point of the curve), the PoW model can reconstruct corrected substitution rates for virus genotypes with increasingly divergent nucleotide sequences.

While the empirical observation of the power-law rate decay has enabled the reconstruction of the timescales of association between some viruses and their hosts,^31^ these approaches are based on using a top-down description of rate decay, which lacks an underlying biological basis. Furthermore, they require the use of multiple internal calibration points in order to estimate timescales. The PoW model does not require such calibrations and does not exhibit substantial rate decay over short timescales (i.e., the flat part of the rate curve) before the fastest-evolving sites have saturated. This enables reliable inference of divergence time over shallow timescales; over such timescales a naive extrapolation of substitution rates using the empirical power-law can produce inaccurate divergence date estimations.

Our mechanistic model allows for a fraction of sites to evolve at different rates due to epistasis or nucleotide biases, requires a minimal number of assumptions, and needs no additional calibration information. This provides, for the first time, a bottom-up model that can account for the empirical observation of the TDRP. The model is compatible with the notion of host-driven constraints on virus evolution,^27^ which represents a special case of the PoW model, but it does not require the constraints to be host driven and is generalizable. Furthermore, we find the substitution rate at the fastest-evolving rate groups in RNA viruses to be 1 to 2 orders of magnitude faster than DNA viruses. This provides RNA viruses with an access to a wider range of sites that can evolve at intermediate substitution rates which, in turn, provides them with more possibilities for epistatic substitutions. We know that this is indeed the case for most RNA viruses due to their compact genomes, overlapping reading frames, and secondary structures.

## STAR★Methods

### Key resources table

**Table:** 

REAGENT or RESOURCE	SOURCE	IDENTIFIER
**Deposited data**		

Sarbecovirus alignments	^34^	https://github.com/plemey/SARSCoV2origins
Hepatitis C virus alignments	This paper	https://github.com/mg878/PoW_model
Foamy virus alignments	^31^	See Additional file 4L in Aiewasakn and Katzourakis^31^
396 viral nucleotide rate estimates	^25^	See Supplemental material, Table S1 in Aiewsakun and Katzourakis^25^

**Software and algorithms**		

MUSCLE	^41^	Version 3.8.425; RRID: SCR_011812
BEAST	^42^	Version 1.10; RRID: SCR_010228
TreeAnnotator	^43^	Version 1.10.4; RRID: SCR_017307
Tracer	^44^	Version 1.7; RRID: SCR_017307
Mathematica	Proprietary	Version 11.0; https://www.wolfram.com/mathematica
RStudio	GNU	Version 4.0.5; RRID: SCR_000432
ggptree	R package	Version 2.4.2
ape	R package	Version 5.5; RRID: SCR_009122
treeio	R package	Version 3.3.2
nleqslv	R package	Version 1.14.4

### Resource availability

#### Lead contact

Further information and requests for resources and datasets should be directed to and will be fulfilled by the Lead Contact, Aris Katzourakis aris.katzourakis@zoo.ox.ac.uk.

#### Materials availability

The list of all used resources is provided in the Key resources table.

### Experimental model and subject details

All the sources of sequence alignments and bioinformatic data used in the analysis are provided in the Key resources table.

#### Sarbecovirus dataset

To minimize the effect of recombination when inferring time-tree phylogenies of Sarbecovirus, we use the putative recombination-free alignment from Boni et al.^34^ with 66 sequences (also called the non-recombinant alignment 3, NRA3).

#### Hepatitis C virus dataset

All coding complete genome sequences of HCV were downloaded from GenBank in May 2019. Those with annotated sample dates were then quality tested (completeness, lack of internal gaps, stop codons, ambiguous bases), and then filtered for sequence similarity to each other. A threshold of 0.2 nucleotide sequence divergence (over the whole genome) was used to extract single examples of each subtype that were dated and supplemented these with 17 further sequences that were the most divergent examples of the same subtype (threshold of 17%). The set was further supplemented with examples of genotypes 1a, 1b and 3a, and references sequences of all subtypes to produce a final alignment of 120 sequences.

#### Foamy virus dataset

We use the manually-curated *pol* nucleotide (3,351 nucleotides) alignments of 14 extant FVs from Aiewsakun and Katzourakis.^25^ The dataset was examined for potential recombination by Aiewsakun and Katzourakis and no evidence for significant recombination was found. Because the viral tree topology is closely aligned with the host phylogeny, we assumed a long history of cospeciation for these viruses and matched the viral tree topology to that of the hosts (enforced certain taxon sets to be monophyletic in the BEAST analysis).

#### R package for the construction of PoW-transformed phylogenies

For heterochronous datasets (i.e., sequence data isolated at different time points), we first infer the mean rate using standard substitution models. We then construct an ultrametric distance tree using either the JC69 or HKY85 substitution models. To convert the distance trees into a PoW-transformed time tree, we first subsample distance trees that are produced by BEAST and convert each of them to time trees using the PoW model (see Equations 5 and 6) assuming that the short-term rate (i.e., flat part of the sigmoid curve) is equal to the median substitution rate inferred from the previous step and that the fastest-evolving rate group (which sets the inflection point) matches with the inferred $\mu_{\max}$ from Table 1 based on the Baltimore group that the virus belongs to. Finally, we use TreeAnnotator to build a consensus tree and find the estimated median and 95% HPD node heights. This approach fully captures the uncertainty that may arise from the substitution model and tree topology. We note that while in the first step we may use substitution models with rate heterogeneity (such as a gamma distribution) and various clock models to infer the short-term rate, in the second step (i.e., constructing ultrametric distance trees) we must use a strict clock (i.e., rate = 1 across all branches) to infer the genetic distance between every pair of sequences.

To further capture the effect of variation in the inferred mean rate (from BEAST) and $\mu_{\max}$ (from geometric least square fit) on the clock model and, ultimately, the PoW-transformed ultrametric distance trees, we can either randomly draw numbers from the posterior rate distribution or any other appropriate statistical distriubtions to find a range of parameter values for the short-term substitution rate, ⟨μ⟩, and $\mu_{\max}$. This enables us to convert each sampled distance tree into a time tree with a unique pair of values for ⟨μ⟩and $\mu_{\max}$.

#### Substitution rate inference of simulated datasets

We simulate a neutral haploid Wright-Fisher population of size $N_{e}$ with L evolving sites under a constant mutation rate μ per site such that every nucleotide (A, C, G, and T) can mutate to any other nucleotide at the same rate μ/3 – mutation rate is equal to substitution rate under neutrality. We then sample from the entire population at two time points with an increasingly wider time gap, $t^{*}$. Initially, we allow the population to evolve for $10N_{e}$ generations before taking the first sample to ensure that neutral coalescent events reach their steady state distribution and that the population, on average, coalesces every $2N_{e}$ generations. We then take the second sample $t^{*}$ generations later and repeat this process 100 times to generate replicate sequences at both time points and run each set of simulations in BEAST 1.10 to estimate the substitution rate (Figure S1). We load the simulated sequences (along with their sampling times) on BEAST and use a strict molecular clock with a continuous-time Markov chain reference prior on substitution rates, a constant population coalescent prior, and a Jukes-Cantor substitution model. For every simulated set, the Markov chain Monte Carlo was run for 10,000,000 steps and parameter convergence was inspected visually.

### Method details

#### Power-law rate decay due to site saturation

For a sequence that has diverged from its ancestor *t* generations ago under a constant and uniform substitution rate μ per site per year, the proportion of pairwise differences, p(t), is given by Tajima and Nei^41^

(Equation 1) $$p\left(t\right)=\alpha \left(1-e^{-\mu t/\alpha }\right)$$

such that α is the maximum proportion of pairwise differences and is given by $\alpha =1-\sum_{i}\pi_{i}^{2}$ where $\pi_{i}$ is the base frequency of the *i*th nucleotide or amino acid. Assuming that d is the ‘true’ genetic distance between a pair of homologous sequences, i.e., d=μt, we can estimate the observed genetic distance, $\hat{d}$, with an observed proportion of pairwise differences, $\hat{p}$, using the Felseinstein’s 1981 substitution model^42^

(Equation 2) $$\hat{d}=\hat{\mu t}=-\alpha_{M}Ln\left\{1-\hat{p}/\alpha_{M}\right\}$$

where $\alpha_{M}$ is the expected saturation frequency set by the substitution model. If the model correctly identifies the saturation frequency, i.e., $\alpha_{M}=\alpha$, Equation 2 accurately predicts the true genetic distance, i.e., $\hat{d}=d$, as long as the divergence time t≪α/μ. As p(t) approaches saturation frequency at $t^{*}\approx \alpha /\mu$, the observed proportion of pairwise differences will be bound by the number of evolving sites. For instance, if the saturation frequency is α=3/4, i.e., a standard Jukes-Cantor substitution model, to distinguish between an observed pairwise difference of ${\hat{p}}^{*}=0.74$ and ${\hat{p}}^{*}=0.741$requires approximately one thousand evolving sites, all evolving at rate μ. Thus, beyond $t^{*}$, the estimated distance, $\hat{d}$, will remain effectively unchanged. In other words, if the pair diverge beyond the saturation point, the inferred rate follows a power-law rate drop with a slope −1 on a log-log plot (see gray curve in Figure S1A).

In the presence of purifying selection and/or amino acid and nucleotide biases, the substitution model in Equation 2 may overestimate the maximum proportion of pairwise differences, i.e., $\alpha_{M}>\alpha$. For instance, if we apply a Jukes-Cantor measure of nucleotide distance to a pair of sequences with a particular site preference that equally favors only two (out of the four) nucleotides, i.e., α=1/2, the proportion of pairwise differences reaches saturation much earlier than what the chosen substitution model would predict. As a result, similar to the previous example ($\alpha_{M}=\alpha$), the estimated rate drops as a power-law with slope −1 (orange curve in Figure S1A) after reaching the saturation point, i.e., $\hat{\mu }\approx \alpha_{M}Ln\left\{\alpha_{M}/\left(\alpha_{M}-\alpha \right)\right\}/t$.

In the opposite extreme, i.e., when $\alpha_{M}<\alpha$, the substitution model underestimates the true saturation frequency. Thus, the observed proportion of pairwise differences surpasses the level predicted by the substitution model, i.e., $\hat{p}/\alpha_{M}≳1$, at which point the estimated substitution rate, $\hat{\mu }\approx \alpha Ln\left\{\alpha /\left(\alpha -\alpha_{M}\right)\right\}/t$, goes to infinity (blue curve in Figure S1A).

#### Saturation of sites in the presence of rate heterogeneity

In the presence of rate heterogeneity, a fraction of sites may evolve at a rate that is much slower (or faster) than some other sites. In principle, this process may involve *M* different rate groups such that each group *i* evolves at rate $\mu_{i}$ and occupies a fraction of sites *m*_*i*_. Thus, the proportion of pairwise differences would be given by

(Equation 3) $$p\left(t\right)=\sum_{i=1}^{M}m_{i}\alpha_{i}\left(1-e^{-\mu_{i}t/\alpha_{i}}\right)$$

such that $\sum_{i}m_{i}=1$ with the mean substitution rate $\left\langle \mu \right\rangle =\sum_{i}m_{i}\mu_{i}$. If we apply a measure of distance based on Equation 2 to an evolutionary process with rate heterogeneity, Equation 3, it can reliably infer the mean substitution rate up to when the sites belonging to the fastest substitution rate, $\mu_{\max}$, reach saturation after $t∼\mu_{\max}^{-1}$ at which point the pattern of time-dependent rate decay emerges and the mean substitution rate gradually plateaus at a value corresponding to the substitution rate of the slowest-evolving sites, $\mu_{\min}$. Once all the rate groups reach saturation at time $t∼\mu_{\min}^{-1}$, the power-law rate decay with slope −1 emerges.

For instance, if the sequence evolution involves two rate groups, i.e., *M = 2*, a fraction of sites *m*_*1*_ may evolve neutrally at rate $\mu_{1}=\mu$ and the remaining sites ($1-m_{1}$) evolve epistatically such that a pair of sites need to mutate simultaneously to recover the wild-type fitness, i.e., $\mu_{2}=\mu^{2}$. Assuming the saturation frequency across all sites is equal and that the model correctly identifies their frequency, i.e., $\alpha_{i}=\alpha_{M}=\alpha$, we can use Equation 2 to recover the expected substitution rate $\left\langle \mu \right\rangle =m_{1}\mu +\left(1-m_{1}\right)\mu^{2}$. As the fast-evolving sites approach the saturation point at $t_{1}\approx \alpha /\mu$, the rate decay emerges and a sharp decline in estimated substitution rate follows while the remaining fraction of sites, $\left(1-m_{1}\right)$, keep accumulating new substitutions at rate $\mu^{2}$, slowing down the speed of the rate decay until those sites also reach saturation at $t_{2}\approx \alpha /\mu^{2}\;$beyond which point the entire genome reaches saturation and the power-law rate decay with slope −1 emerges. Figure S1B shows that as the proportion of slow-evolving sites increases, the mean substitution rate goes down and the slope of the time-dependent rate decay becomes less steep.

Although our focus so far has been on the saturation of pairwise differences and how it can create a time-dependent rate effect, the same holds true when tracking the evolutionary changes of a large number of sequences through time. Using a standard Jukes-Cantor substitution model on a set of simulated sequences, both in the absence and presence of rate heterogeneity, we can recreate similar patterns of time-dependent rate decay and show that, over longer timescales, i.e., when the divergence time between two populations is much longer than the typical coalescent times, the variation in inferred substitution rates is dominated by the saturation along the longest (internal) branch connecting the two populations (Figures S1C–S1H). We also find that, over short timescales, systematic under-estimation of the Time to the Most Recent Common Ancestor (TMRCA) results in inflated substitution rate estimates (Equation 7).

#### Saturation of sites under the PoW model

Under the PoW model, the virus substitution rate is categorised into M discrete rate classes such that there is a fixed incremental difference between any two consecutive rate groups, $\mu_{i+1}=\text{Δ}M\mu_{i}$, with a common ratio ΔM. Rate groups range from those evolving the fastest, at rate $\mu_{\max}$, to the ones evolving at the host substitution rate, $\mu_{\min}$. The fraction of sites, $m_{i}$, in each rate group i, evolving at rate, $\mu_{i}$, is an exponentially distributed number, $m_{i}=Ce^{\lambda i}$, where C is the normalization factor, $C=1/\sum_{j=1}^{M}e^{\lambda j}$, and the exponent coefficient, λ, sets the tendency of sites to be either mostly slowly (λ<0) or rapidly (λ>0) evolving. Given a fixed common ratio between consecutive rate groups, the substitution rate at the fastest-evolving sites can be determined by finding the total number of rate groups, M, which, in turn, sets the inflection point for when the time-dependent rate decay emerges. Once the fastest-evolving sites reach the saturation point, other rate groups that evolve more slowly (e.g., via epistatic and compensatory substitutions), saturate chronologically as the time span of rate measurement, t, increases. This chronological saturation effect continues until the inferred rate decays to the host substitution rate, $\mu_{\min}$. Therefore, the time-dependent rate curve, according to the PoW model is given by

(Equation 4) $$\hat{\mu }\left(t\right)=-\alpha_{M}Ln\left(1-\frac{1}{\alpha_{M}}\sum_{i=1}^{M}\alpha m_{i}\left(1-e^{-\mu_{i}t/\alpha }\right)\right)/t$$

where the observed genetic distance between the derived and ancestral sequences, $\hat{d}=\hat{\mu }t$, is proportional to the time span of rate measurement, t. While over short timescales, i.e., $t\ll 1/\mu_{\max}$, several methodological (e.g., internal node calibration errors) and biological (e.g., purifying selection) artifacts may inflate the substitution rate estimates in viruses (i.e., such that $\hat{\mu }\left(t\right)$ underestimates the inferred rates), over longer time-scales (i.e., typically after a few years) the rate estimates are expected to be closer to the mean (short-term) substitution rate, $\left\langle \mu \right\rangle =\sum_{i=1}^{M}m_{i}\mu_{i}$. Over such timescales, there is no site saturation, i.e., the rate curve is flat, and the inferred short-term substitution rates can be reliably used to estimate divergence times between samples without significant influence from site saturation. We also note that the finer the gap between consecutive rate groups, ΔM, gets, the more accurate the estimated rate curve becomes. However, typically, less than 50 rate groups (i.e., M<50) is sufficient for all predictions. For a fixed ΔM, the exponent coefficient, λ, together with the number of rate groups, M, are the two free parameters of the PoW model which set the short-term, ⟨μ⟩, and maximum, $\mu_{\max}$, substitution rates for any given dataset. While Equation 4 assumes that the saturation frequency, α, across all sites is the same, there can be instances where, due to site preferences, some mutations do not appear at certain positions in the virus genome. This can result in a reduction in saturation frequency at those sites (i.e., α<3/4). However, to avoid overparametrizing the model, in the absence of sufficient data, we assume identical saturation frequencies across all sites.

#### Distance tree transformation using the PoW model

Equation 4 allows for a one-to-one map between the inferred genetic distance and divergence time. Therefore, by estimating the genetic distance (in units of substitutions), $\hat{d}$, between any pair of sequences under a JC69 substitution model (i.e., $\alpha_{M}=\alpha =3/4$) we can solve Equation 5 to find the divergence time, *t*, since the common ancestor of each pair using the PoW model.

(Equation 5) $$\hat{d}=-\frac{3}{4}Ln\left(1-\sum_{i=1}^{M}m_{i}\left(1-e^{-4\mu_{i}t/3}\right)\right)$$

More generally, we can apply other, more complex, substitution models to infer the genetic distance between pairs of sequence. For instance, under the Tamura-Nei substitution model (TN93) where there is an analytically tractable formula for distance,^43^ the PoW-transformed equation to find divergence time, *t*, is given by

(Equation 6) $$\hat{d}=\frac{2\pi_{T}\pi_{C}}{\pi_{Y}}\left(a_{1}-\pi_{R}b\right)+\frac{2\pi_{A}\pi_{G}}{\pi_{R}}\left(a_{2}-\pi_{Y}b\right)+2\pi_{Y}\pi_{R}b,$$

$${\overset{⌢}{k}}_{1}=\frac{a_{1}-\pi_{R}b}{\pi_{Y}b},$$

$${\overset{⌢}{k}}_{2}=\frac{a_{2}-\pi_{Y}b}{\pi_{R}b},$$

where

$$a_{1}=-Ln\left(1-\frac{\pi_{Y}S_{1}}{2\pi_{T}\pi_{C}}-\frac{V}{2\pi_{Y}}\right),$$

$$a_{2}=-Ln\left(1-\frac{\pi_{R}S_{2}}{2\pi_{A}\pi_{G}}-\frac{V}{2\pi_{R}}\right),$$

$$b=-Ln\left(1-\frac{V}{2\pi_{Y}\pi_{R}}\right),$$

$$S_{1}=2\pi_{T}p_{TC}\left(t\right),$$

$$S_{2}=2\pi_{A}p_{AG}\left(t\right),$$

$$V=2\pi_{T}p_{TA}\left(t\right)+2\pi_{T}p_{TG}\left(t\right)+2\pi_{C}p_{CA}\left(t\right)+2\pi_{C}p_{CG}\left(t\right),$$

such that $k_{1}$ and $k_{2}$ are the two different types of transition rates (transversions are all assumed to occur at the same rate) according to the TN93 model, $\pi_{i}$ is the nucleotide equilibrium base frequency, and $p_{ij}\left(t\right)$ is the transition probability (not to be confused with transition rate) to go from nucleotide *i* to *j* according to the PoW model, (i,j)={A,C,G,T}. While the equilibrium base frequencies and transition rates can be found numerically from the phylogenetic analysis using BEAST, the transition probabilities are found from the eigenvalues of the transition matrix $P\left(t\right)=\left\{p_{ij}\left(t\right)\right\}=e^{Qt}$ (see Ho et al.^43^ for more details on the calculations). For example, according to the PoW model, $p_{TA}\left(t\right)=\sum_{i=1}^{M}m_{i}\pi_{A}\left(1-e^{-\beta_{i}t}\right)$, where $\beta_{i}$ is the transversion rate from rate group *i*. We can also find a relationship between the average substitution rate per rate group, $\mu_{i}$, and $\beta_{i}$ which is given by

$$\mu_{i}=2\beta_{i}\left(k_{1}\pi_{T}\pi_{C}+k_{2}\pi_{A}\pi_{G}+\pi_{Y}\pi_{R}\right).$$

Similarly, Equation 6 can be used for the PoW transformation to be applied to a wider range of substitution models such as the HKY85 substitution model (i.e., $k_{1}=k_{2}$).

#### Saturation of sites for simulated datasets

In Figures S1C–S1H, we recreate the time-dependent pattern of rate decay both in the absence and presence of rate heterogeneity across sites, using a standard substitution model on simulated data. We find that while the inferred substitution rates exhibit a power-law rate decay with slope −1 over longer time intervals (see Figures S1C and S1D), the inferred TMRCAs tend to be overestimated with a similar (inverse) power-law trend, i.e., $\hat{t}∼1/\hat{\mu }$ (see Figures S2E and S2F). We also find an unexpected time-dependent rate effect over short timescales. This occurs when the observation gap, $t^{*}$, is much shorter than the expected coalescent time of the population, i.e., $t^{*}∼2N_{e}$. This also results in the underestimation of true TMRCAs which systematically makes worse predictions for higher substitution rates. The expected rate curves (dashed lines shown in Figures S2C and S2D) can be approximated by replacing p(t) from Equation 1 into $\hat{p}$ from Equation 2 which is given by

(Equation 7) $$\hat{\mu }\left(t^{*}\right)\approx -\alpha_{M}Ln\left\{1-\frac{1}{L\alpha_{M}}\left\lfloor L\sum_{i=1}^{M}m_{i}\alpha \left(1-e^{-\mu_{i}\left(t^{*}+2N_{e}\right)/\alpha }\right)\right\rfloor \right\}/\left(t^{*}+T\right)$$

such that ⌊.⌋ is the floor function which represents the finite size effect of having L evolving sites on saturation frequency. The mean divergence time between the two populations is approximately $t\approx t^{*}+2N_{e}$ and the inferred divergence time is $\hat{t}\approx t^{*}+T$ – this resembles the mis-calibration effects reported elsewhere (see Equation 2 in Ho et al.^44^). The reason why Equation 4 only works as an approximate is that the median inferred TMRCA from simulation results, T, also varies with respect to observation gap $t^{*}$ (see Figures S1E and S1F). However, for $t^{*}\gg 2N_{e}$, the variation in T becomes negligible compared to $t^{*}$and only has second-order effects on inferred substitution rates. Figures S2G and S2H show the tree topology under the two extremes, $t^{*}\ll 2N_{e}$ and $t^{*}\gg 2N_{e}$, respectively. It indicates that, over long timescales, the time-dependent rate effects are dominated by the very long (and saturated) branch connecting the two populations that are $t^{*}$ generations apart. As a result, the decay dynamics looks very similar to the analytical results in Figures S1A and S1B where we estimate the substitution rate between a pair of sequences separated by a very long branch.

### Quantification and statistical analysis

The Method details provide in-depth descriptions of the quantifications and statistical analyses used in this manuscript.

## Data Availability

All datasets and codes required to reproduce the analyses are available at https://github.com/mg878/PoW_model.

## Supplemental information

Document S1. Figures S1–S3 and Table S1

Document S2. Article plus supplemental information
