# Supplementary material for: A mechanistic evolutionary model explains the time-dependent pattern of substitution rates in viruses
Source: Curr Biol. 2021 Nov 8;31(21):4689–4696.e5. doi: 10.1016/j.cub.2021.08.020 (PMC8585505; doi:10.1016/j.cub.2021.08.020)
Supplement: Document S1. Figures S1–S3 and Table S1 [file mmc1.pdf]

Current Biology, Volume *31*

Supplemental Information

A mechanistic evolutionary model explains the time-dependent pattern of substitution rates in viruses

Mahan Ghafari, Peter Simmonds, Oliver G. Pybus, and Aris Katzourakis

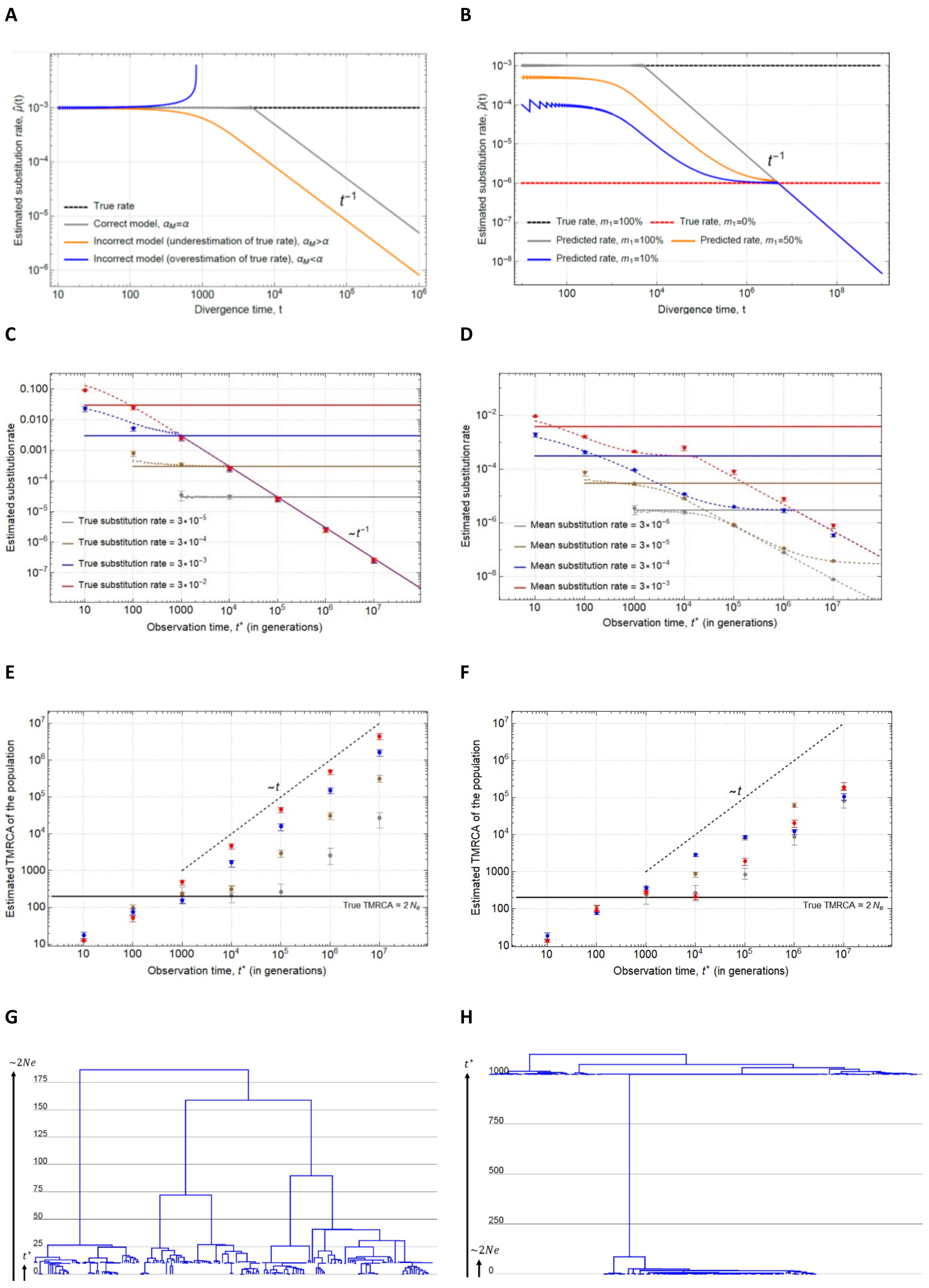

Figure S1. Estimated substitution rates, time to the most recent common ancestors, and phylogenetic trees of simulated datasets under different substitution rates. Related to STAR Methods and Figure 1.

(A) Estimating the substitution rate $\mu = 10^{-3}$ (black dashed line) using **Equation S2** for a pair of sequences that have diverged from each other t generations ago under the correct substitution model (gray), a model that over-estimates the true saturation frequency (orange), α, and a model that under-estimates the true saturation frequency (blue). **(B)** Estimating the expected substitution rate, $< \mu >$, when a fraction m_1 of sites evolve at rate $\mu_1 = 10^{-3}$ (black dashed line) and the remaining fraction, $1 - m_1$, at rate $\mu_2 = 10^{-6}$ (red dashed line). The expression t^{-1} in both plots shows the dominating term in rate decay with respect to divergence time, t, corresponding to slope -1 on the graphs. (**C**) A population of size N_e evolving neutrally with $L_n = 100$ sites evolving at the same rate, μ, and (**D**) a model with rate heterogeneity across sites such that $L_1 = 100$ sites evolve at rate μ and the remaining $L_2 = 900$ sites at rate μ^2. The rate is estimated as a function of observation gap, t^*, between when the first and second sampling point from the population. Dashed lines show the theoretical prediction according to **Equation S6** and solid lines show the mean (expected) rates used for the simulations. (**E**) and (**F**) show the estimated TMRCA for the first group of sampled sequences, i.e. at $t^* = 0$, and solid lines show the mean TMRCA according to neutral theory. The rates and TMRCAs are estimated using BEAST under a strict clock assumption. Dots represent the median values taken from 100 independent runs and error bars show the interquartile region. (**G**) and (**H**) show the maximum clade credibility trees for one simulation run corresponding to $\mu = 3 \times 10^{-5}$ when the observation gap is $t^* = 10,\ t^* \ll 2N_e$ and $t^* = 1000,\ t^* \gg 2N_e$, respectively.

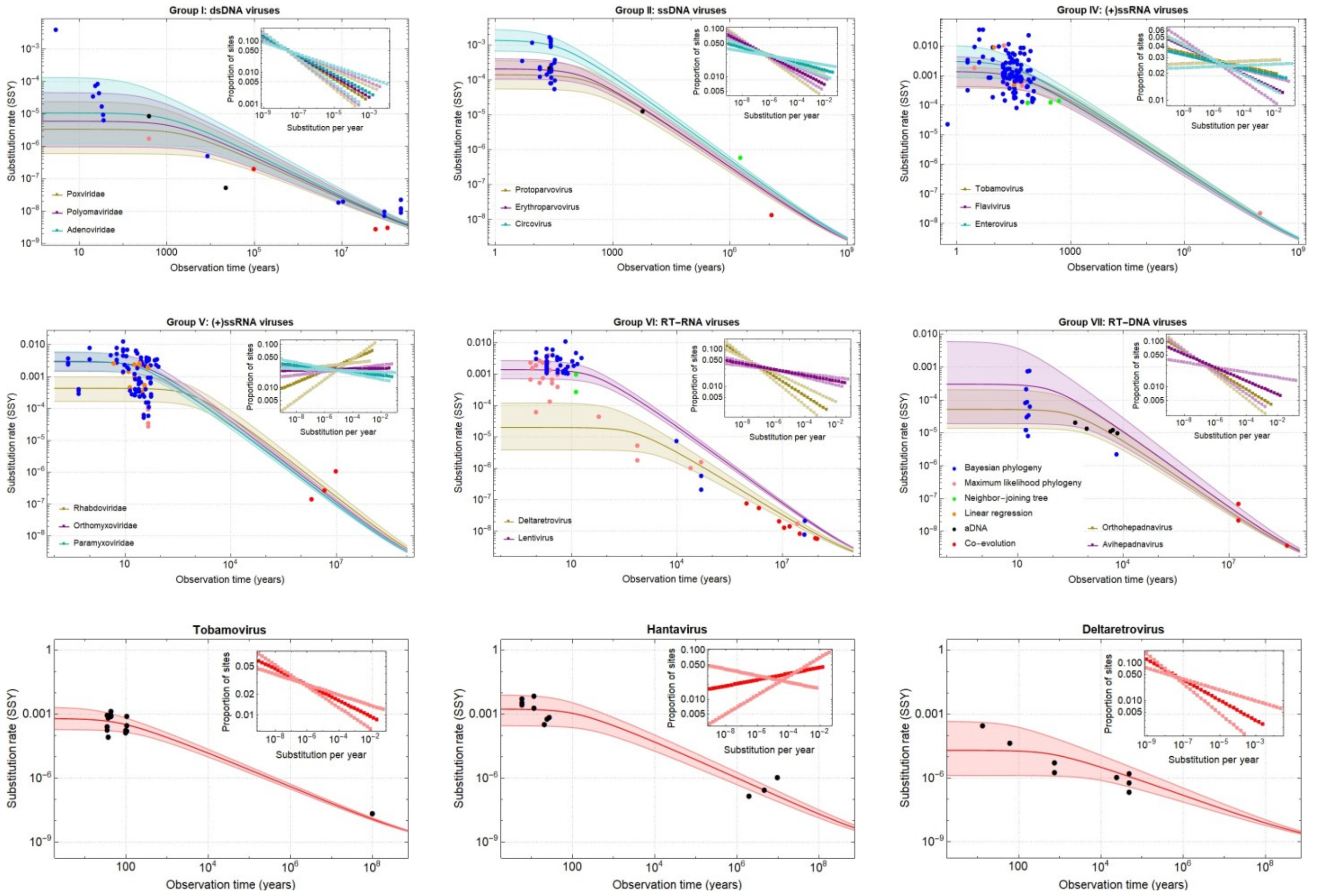

Figure S2. Estimated time-dependent rate curves for each viral group and three selected genera according to the PoW model and their corresponding distribution of rate groups (inset). Related to Figure 2.

The first two rows show two or three distinct mean substitution rates (coloured in gold, purple, and blue) that are selected for each virus family to estimate rate curves according to the PoW model. The bottom row shows the same rate curve estimations for Tobamoviruses with $\langle \mu \rangle = 0.6(0.2-2)\times 10^{-3}$ SSY and $\mu_{max} = 3(1-6)\times 10^{-2}$ SSY, Hantaviruses with $\langle \mu \rangle = 2(0.3-8)\times 10^{-3}$ SSY and $\mu_{max} = 2(0.6-4)\times 10^{-2}$ SSY, and Deltaretroviruses with $\langle \mu \rangle = 2(0.1-50)\times 10^{-5}$ SSY and $\mu_{max} = 3(2-30)\times 10^{-3}$ SSY. These genera are selected as they have the widest timespan of rate measurement. The solid lines show the best fit and shaded areas the 95% confidence interval ($\Delta M = 1.58$ and $\alpha_M = \alpha = 3/4$).

A

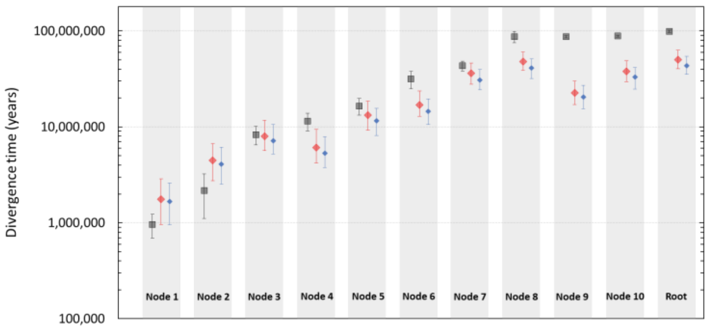

B

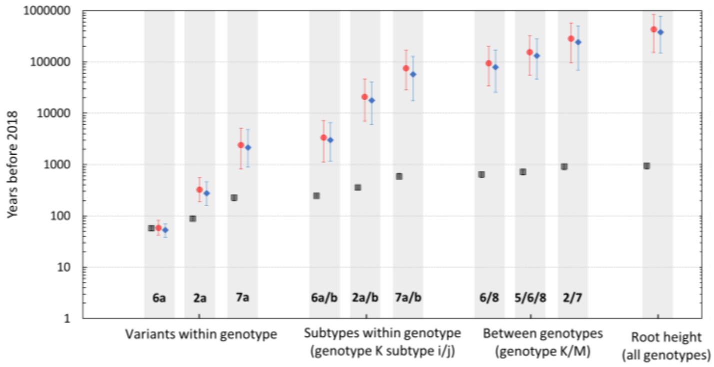

C

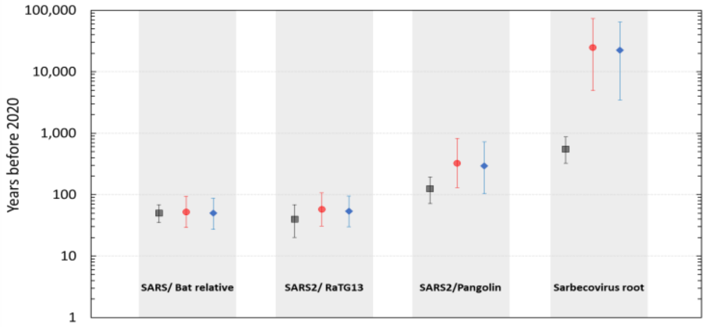

Figure S3. Estimated divergence times for FV, HCV, and sarbecovirus datasets using standard substitution models and PoW transformation with uncertainty in the underlying clock model. Related to STAR Methods and Figure 3.

Same legend as **Figure 3**. (**A**) Estimated divergence times between labelled internal nodes of FV (see **Figure 3**). For the PoW transformation using the HKY85 and JC69 substitution models, the short-term substitution rate, $\langle \mu \rangle$, was randomly sampled from the Normal distribution, $N(\text{mean} = 6.1\times10^{-6}, \text{stdev} = 2.0\times10^{-6})$. (**B**) For HCV, we compare the results between a standard HKY+G model and two POW-transformed estimates using the HKY85 and JC69 substitution models. The short-term substitution rate, $\langle \mu \rangle$, was randomly sampled from the posterior rate distribution of the standard HKY+G model. (**C**) Similarly, for Sarbecovirus, we compare the results between a standard HKY+G model and two POW-transformed estimates using the HKY85 and JC69 substitution models. The short-term substitution rate, $\langle \mu \rangle$, was randomly sampled from the posterior rate distribution of the standard

HKY+G model. In all the three datasets, the fastest-evolving rate group, $\mu_{\max}$, was sampled from the Normal distribution, $N(\text{mean} = 3.65 \times 10^{-2}, \text{stdev} = 5.0 \times 10^{-3})$.

Viral group	Virus family/genus used for calibration	Short-term substitution rate, $\langle \mu \rangle$	Fastest rate group, μ_{max}
Group I	Poxviridae	$0.3(0.06-2)\times 10^{-5}$	$0.6(0.2-3)\times 10^{-3}$
	Polyomaviridae	$0.6(0.1-5)\times 10^{-5}$	$1(0.3-4)\times 10^{-3}$
	Adenoviridae	$1(0.1-10)\times 10^{-5}$	$2(0.3-10)\times 10^{-3}$
Group II	Protoparvovirus	$1(0.6-4)\times 10^{-4}$	$0.6(0.4-1)\times 10^{-2}$
	Erythroparvovirus	$2(1-4)\times 10^{-4}$	$1(0.6-2)\times 10^{-2}$
	Circovirus	$10(7-300)\times 10^{-4}$	$4(3-6)\times 10^{-2}$
Group IV	Tobamovirus	$0.4(0.9-2)\times 10^{-3}$	$2(1-3)\times 10^{-2}$
	Flavivirus	$1(0.4-5)\times 10^{-3}$	$4(2-10)\times 10^{-2}$
	Enterovirus	$3(0.9-10)\times 10^{-3}$	$6(3-10)\times 10^{-2}$
Group V	Rhabdoviridae	$0.4(0.2-1)\times 10^{-3}$	$0.3(0.2-0.4)\times 10^{-2}$
	Orthomyxoviridae	$3(1-6)\times 10^{-3}$	$4(3-6)\times 10^{-2}$
	Paramyxoviridae	$3(2-6)\times 10^{-3}$	$6(4-10)\times 10^{-2}$
Group VI	Deltaretrovirus	$0.02(0.004-0.1)\times 10^{-3}$	$0.3(0.06-1)\times 10^{-2}$
	Lentivirus	$1(0.7-3)\times 10^{-3}$	$4(3-6)\times 10^{-2}$
Group VII	Orthohepadnavirus	$5(1-20)\times 10^{-5}$	$4(2-10)\times 10^{-3}$
	Avihepadnavirus	$30(2-600)\times 10^{-5}$	$20(2-100)\times 10^{-3}$

Table S1. Estimated short-term substitution rate and fastest-evolving rate group across 6 viral groups. Related to Table 1.

Only short-term rate estimates (measured over time scales of <100 years) – along with the long-term rate estimates (>100 years) from the entire data set – from a particular virus family or genus (rather than the entire viral group) is used for rate calibration in the PoW model. Parentheses correspond to 95% confidence intervals.
